# Mise en place d´un système de management de la qualité dans une unité de stérilisation centralisée: expérience de l´Hôpital Tahar Sfar de Mahdia en Tunisie

**DOI:** 10.11604/pamj.2021.39.287.25653

**Published:** 2021-08-31

**Authors:** Mohamed Hedi Ben Cheikh, Maher Teka, Mahmoud Fhal, Kaouther Zribi, Ali Majdoub

**Affiliations:** 1 Laboratoire de Développement des Médicaments (LR12ES09), Université de Monastir, Faculté de Monastir, Rue Ibn Sina, 5000, Monastir, Tunisie,; 2 Service de Pharmacie, Hôpital Taher Sfar de Mahdia, Djbal Dar Ouaja, 5100, Mahdia, Tunisie,; 3 Service d´Orthopédie, Hôpital Taher Sfar de Mahdia, Djbal Dar Ouaja, 5100, Mahdia, Tunisie,; 4 Service de Pharmacie, Centre de Maternité et de Néonatologie de Monastir, 5000, Monastir, Tunisie,; 5 Service d´Anesthésie Réanimation, Hôpital Taher Sfar de Mahdia, Djbal Dar Ouaja, 5100, Mahdia, Tunisie

**Keywords:** Assurance qualité, stérilisation, audit, Quality assurance, health care, sterilization, management audit

## Abstract

La réglementation de la stérilisation hospitalière en Tunisie recommande la mise en place d´un système d´assurance qualité. L´objectif de ce travail était de dresser l´état des lieux dans une unité de stérilisation afin d´évaluer le respect des bonnes pratiques et d´explorer les opportunités d´amélioration. Une étude prospective a été réalisée en 2019 dans l´unité de stérilisation du centre hospitalo-universitaire de Mahdia. Deux audits internes, réalisés dans les mêmes conditions, ont été menés à un an d´intervalle. Le premier audit a permis de cerner les défaillances et les dysfonctionnements et la planification d´un plan d´actions. L´impact des mesures entreprises a été évalué par un deuxième audit. La collecte des données a été effectuée par observation directe des ressources et des pratiques existantes. Le taux de conformité a été calculé en tenant compte des critères conformes et des critères applicables. Les résultats du premier audit ont révélé un taux de conformité de l´ordre de 28,1%. L´analyse des écarts a permis de dégager 5 axes d´améliorations dont principalement la mise en place d´un système documentaire et de management de la qualité. Au total, nous avons établi 14 documents correspondant aux processus managériaux, 26 aux processus opérationnels et 41 aux processus supports. Les actions mises en place ont permis d´atteindre un taux de conformité de 60,4%. L´approche adoptée pour la mise à niveau de l´unité de stérilisation a permis de standardiser les pratiques et d´assurer la traçabilité tout au long du processus de stérilisation.

## Abstract (en)

In Tunisia, Hospital sterilization guidelines recommend the establishment of a quality assurance system. The purpose of this study is to give an overview of the situation in a sterilization unit in order to assess the adherence to good practice criteria and to identify opportunities for improvement. We conducted a prospective study in the sterilization unit of the Hospital Tahar Sfar, Mahdia in 2019. Two internal audits were conducted under the same conditions and were carried out one year apart. The first audit identified failures and malfunctions and the outlining of an action plan. The impact of the measures undertaken was tested using a second audit. Data collection was carried out by direct observation of the existing resources and practices. Compliance rate was calculated taking into account compliant criteria and applicable criteria. The results of the first audit revealed a compliance rate of around 28.1%. The analysis used to observe deviations made it possible to identify 5 axes of improvement, in particular the implementation of a system of documentation and a quality management system. In total, we prepared 14 documents related to the managerial processes, 26 to the operational processes and 41 to the support processes. The actions put in place enabled to achieve a compliance rate of 60.4%. The approach taken to upgrade sterilization processes made it possible to standardize them while ensuring traceability.

## Introduction

L´utilisation de dispositifs médicaux réutilisables (DMR) dont le processus de retraitement est défaillant est une source d´infections liées aux soins [1]. Dans les années 90, après la transmission de *Mycobacterium xenopic* à des patients dans une clinique française suite à un non-respect des règles de retraitement du matériel réutilisable, une attention particulière a été portée aux activités de stérilisation dans les établissements de soins [2].

En Tunisie, l´Agence Nationale de Contrôle Sanitaire et Environnemental des Produits «ANCSEP» a mené en 2011 une enquête de grande envergure pour évaluer les pratiques de traitement des DMR dans les structures sanitaires publiques et privées [3]. Les résultats obtenus ont révélé un niveau de sécurité critique, ce qui a suscité les autorités compétentes à entreprendre des mesures d´urgence, notamment l´élaboration d´un guide de bonnes pratiques de traitement des dispositifs médicaux réutilisables en 2013 [4] et la création d´un comité technique de stérilisation qui a pour principale mission la mise à niveau de la stérilisation dans les établissements de santé [5]. Ces décisions ont été soutenues par la promulgation de la circulaire 60 de l´année 2013 remplacée par la circulaire N°8 du 29 janvier 2015 portant organisation des services de stérilisation des dispositifs médicaux dans les établissements sanitaires publics et privés [6]. Cette dernière a exigé l´abandon définitif des techniques de stérilisation par la chaleur sèche. Elle recommande l´autoclavage comme méthode de choix, la centralisation de l´activité et la mise en place un système d´assurance qualité dans un délai de 5 ans.

À l´expiration de cet ultimatum, nous avons envisagé de dresser l´état des lieux dans une unité de stérilisation centralisée d´un hôpital universitaire. En évaluant le respect des bonnes pratiques et l´application de la circulaire en vigueur, nous pourrons explorer des opportunités d´amélioration et mettre en œuvre des actions de mise à niveau.

## Méthodes

Durant l´année 2019, une étude prospective a été réalisée dans l´unité de stérilisation centralisée de l´hôpital Tahar Sfar de Mahdia. Il s´agit d´un centre hospitalo-universitaire d´environ 460 lits et disposant d´un plateau technique composé de 14 salles opératoires. L´unité, qui assure la stérilisation du matériel réutilisable des différentes unités de soins (environ 4000 cycles/an), disposait de 3 autoclaves à double portes d´une capacité totale de 20 paniers DIN (un panier DIN correspond à une dimension standardisée de 600 x 300 x 300 mm pour l´autoclave).

Étant donné que la démarche qualité est une approche systémique faisant appel à des outils adaptés, la méthodologie adoptée était inspirée de la roue de Deming. Afin d´atteindre les objectifs fixés, deux audits internes à une année d´intervalle ont été réalisés par une équipe multidisciplinaire. Le premier audit a permis de dresser l´état des lieux et de cerner les éventuelles défaillances et dysfonctionnement. L´analyse des écarts relevés a servi d´identifier des axes d´amélioration et de planifier un plan d´actions. L´impact des mesures entreprises a été évalué par un deuxième audit, réalisé dans les mêmes conditions.

L´instrument de mesure utilisé était la grille développée par la Société Suisse d´Hygiène Hospitalière [7]. Cette dernière, basée sur le référentiel «bonnes pratiques de retraitement des dispositifs médicaux destinées aux établissements de soins» [8], comportait 207 critères d´observation regroupés dans 10 rubriques (Tableau 1). Pour chaque critère, 3 modalités ont été envisagées: conforme (C), non conforme (NC) ou non applicable (NA).

**Tableau 1:** nombre de critères évalués par rubrique dans la grille utilisée

Rubriques	Nombre de critères
Périmètre d'application	5
Principaux documents de référence	6
Système de management de la qualité	21
Responsabilités	11
Ressources	67
Réalisation du produit	8
Retraitement des dispositifs médicaux	73
Maitrise des dispositifs de surveillance et de mesure	5
Stérilisation pour des tiers	7
Instruments chirurgicaux en prêt	4
**Total**	**207**

La collecte des données a été effectuée par observation directe des ressources et des pratiques existantes. Les données recueillies ont été saisies et exploitées par Office Microsoft Excel 2010. Le taux de conformité a été calculé en divisant le nombre des critères conformes sur celui des critères applicables.

## Résultats

**Figure 1:**
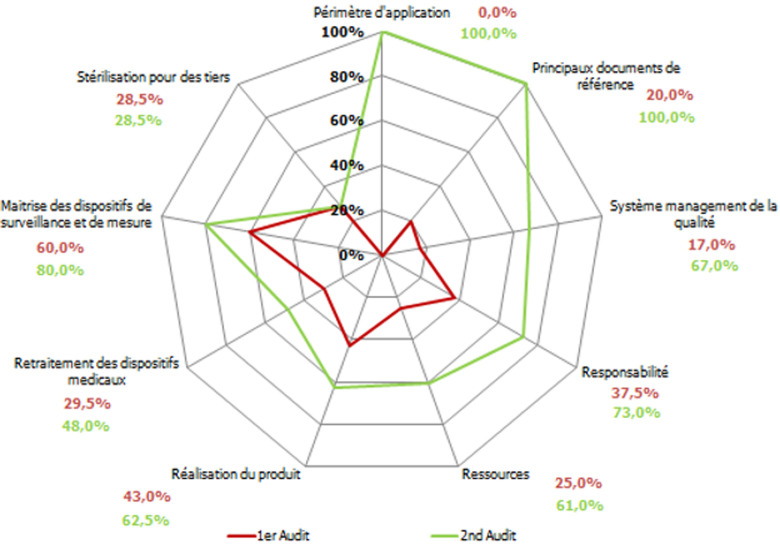
comparaison des résultats des deux audits

**Résultats du premier audit:** les résultats du premier audit ont révélé 72 critères non applicables dans l´unité siège de l´étude. Le taux de conformité du processus de stérilisation par rapport au référentiel était de 28,1%. A l´exception de la rubrique «maitrise des dispositifs de surveillance et de mesure», toutes les autres présentaient des taux de conformité inférieurs à 50% (Figure 1). Les rubriques les plus défaillantes étaient celle du «système management de la qualité» (16,7%) et celle des «principaux documents de référence» (20%).

La rubrique «ressources» présentait un taux de conformité de 25%. Elle incluait les sous rubriques «matériel» (31%), moyens de prévention des infections et protection du personnel» (20%), «locaux» (18%) avec des sous rubriques à 0%: «ressources humaines», «services supports», «eau», «air médical» et «air ambiant». La rubrique «retraitement des dispositifs médicaux», quant à elle, avait un taux de conformité de 29,5% avec des conformités de 10% pour le nettoyage, 20% pour le conditionnement et 36% pour la stérilisation proprement dite.

**Figure 2:**
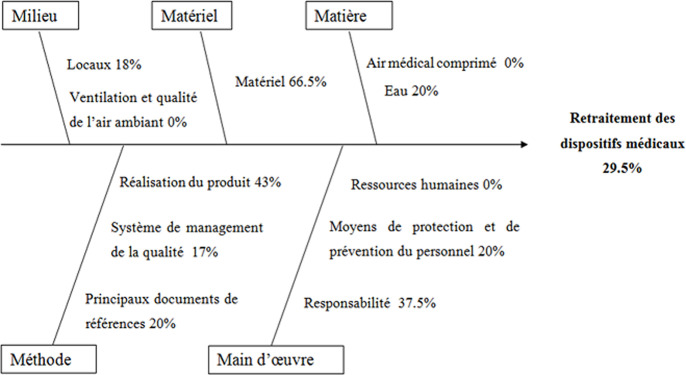
diagramme d'Ishikawa

**Analyse des non-conformités et mise en place du plan d´actions:** le faible taux de conformité de la rubrique «retraitement des dispositifs médicaux» était imputé à des défaillances au niveau des ressources et du système de management de la qualité (SMQ). En effet, une analyse plus approfondie des causes des dysfonctionnements effectuée par le diagramme d´Ishikawa a permis de schématiser cette relation (Figure 2). Ainsi, cinq axes d´amélioration ont été définis: réaménagement des locaux; recrutement et formation du personnel; acquisition, qualification et entretien des équipements; mise en place d´un système de management de la qualité et sécurisation du circuit des DMR.

Compte tenu des contraintes de faisabilité, de temps et de moyens, notre intervention s´est essentiellement focalisée sur la mise en place d´un SMQ en adoptant la démarche suivante: *Action 1:* collecte et analyse des documents réglementaires et normatifs qui régissent l´activité de la stérilisation; *Action 2:* établissement de la cartographie du processus de stérilisation en tenant compte des particularités de l´hôpital de Mahdia; *Action 3:* structuration de l´organisation de l´unité et rédaction des fiches de fonctions; et *Action 4:* élaboration du système documentaire et mise en place du SMQ.

**Collecte et analyse des documents réglementaires et normatifs:** la collecte des documents législatifs et normatifs règlementant l´activité de stérilisation hospitalière a abouti à la création d´un dossier actualisé regroupant les références nationales suivantes: circulaire N°8 du 29 janvier 2015 portant sur l´organisation des services de stérilisation des dispositifs médicaux dans les établissements sanitaires publics et privés [6]. Guide des bonnes pratiques de traitement des dispositifs médicaux réutilisables, version 2013, élaboré par l´Agence Nationale de Contrôle Sanitaire et Environnemental «ANCSEP» [4]. D´autres documents traitant la stérilisation à l´échelle internationale ont été examinés. En plus des normes en vigueur, la liste non exhaustive regroupait les bonnes pratiques en matière de stérilisation des dispositifs médicaux, les guides pour la validation du nettoyage et de la désinfection ainsi que pour le choix des désinfectants.

**Figure 3:**
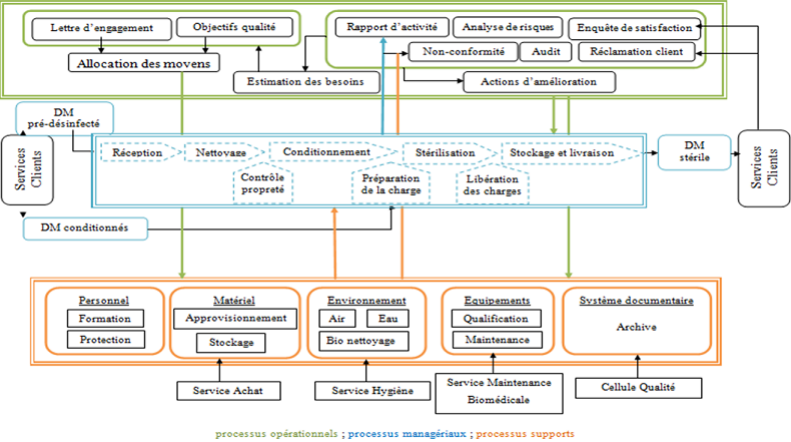
cartographie du processus de stérilisation

**Élaboration de la cartographie du processus de stérilisation:** en tenant compte des pratiques existantes dans l´hôpital, l´analyse approfondie de la documentation de référence (règlementation, normes et recommandations) a permis d´élaborer la cartographie du processus de stérilisation (Figure 3). Cette dernière a fourni une vue d´ensemble sur le circuit de traitement du matériel réutilisable, y compris les flux et les différentes relations entre les produits et les services. En effet, cette cartographie s´appuie sur 3 catégories de processus: les processus opérationnels décrivent l´ensemble des opérations et des contrôles effectués depuis la réception du matériel souillé jusqu´à la livraison du matériel stérile; les processus managériaux décrivent la politique de l´administration, l´organisation et les modalités de contrôle et de surveillance; et les processus supports décrivent les ressources nécessaires au bon fonctionnement de l´unité de stérilisation.

**Structuration de l´organisation de l´unité de stérilisation:** afin de répondre aux exigences structurelles des référentiels, nous avons défini l´organisation hiérarchique de l'unité ainsi que les taches, les rôles et les responsabilités des différents intervenants. À cet effet, un organigramme a été élaboré et des fiches de fonctions décrivant les compétences exigées et les missions attribuées ont été rédigés et validés pour: le pharmacien responsable de l´unité; le surveillant de l´unité; le responsable d´assurance qualité; les agents de stérilisation; et les ouvriers de l´unité de stérilisation.

**Élaboration du système documentaire et mise en place du système management de la qualité:** au total, nous avons élaboré 81 documents qualité dont 6 documents organisationnels, 26 procédures, 22 instructions et 27 formulaires. En fonction de la cartographie du processus de stérilisation, ces documents ont été classés en fonction de leur type de processus: les documents correspondant aux processus managériaux (N=14) ont été élaborés en collaboration avec la direction et la cellule qualité en tenant compte des exigences des services clients; les documents correspondant aux processus opérationnels (N=26) ont été élaborés conformément aux recommandations des bonnes pratiques et en prenant en considération les ressources disponibles et les particularités de l´unité existante; et les documents correspondant aux processus supports (N=41) ont été élaborés en concertation avec les services annexes, notamment ceux de l´hygiène et de sécurité, de la maintenance civile et biomédicale. Afin de mettre en place ce système documentaire, un plan de formation pour l´ensemble du personnel de l´unité a été établi. Ainsi, des séances de formation portant sur l´hygiène et les bonnes pratiques de retraitement des dispositifs médicaux notamment l´importance de la traçabilité ont été programmées et dispensées de façon régulière.

**Résultats du second audit:** les résultats du second audit ont révélé une réduction des critères non applicables (63 versus 72). La répartition des cotations pour chaque rubrique est décrite dans la Figure 1. À l´exception de la rubrique «stérilisation pour des tiers», toutes les autres rubriques ont connu des améliorations de leurs taux de conformité. Sachant que deux rubriques ont atteint 100% de conformité («périmètre d´application» et «principaux documents de référence»), les rubriques «maitrise des dispositifs de surveillance et de mesure» et «responsabilité» ont dépassé un taux de conformité de 70%. Par ailleurs, les rubriques dont le taux de conformité a connu une évolution marquante étaient «système management de la qualité (+50%) et «réalisation du produit» (+20%). Au total, 8 rubriques ont connu une évolution de leurs taux de conformité. Le taux global de conformité du processus de stérilisation par rapport au référentiel était de 60,4%.

## Discussion

En se référant aux exigences de la circulaire N°8 de l´année 2015 portant organisation des services de stérilisation des dispositifs médicaux dans les établissements sanitaires publics et privés et renforcement de leur qualité et efficacité, le CHU de Mahdia présentait des forces et des faiblesses. Le premier point fort était la centralisation de l´activité de stérilisation au sein des mêmes locaux. Cette disposition était présentée par Bahri *et al*. [3], lors d´un audit mené dans deux établissements hospitaliers tunisiens en 2014, comme le seul moyen de garantir la qualité du processus de stérilisation. Par ailleurs, certaines unités de soin au sein de l´hôpital continuaient les phases de nettoyage et de conditionnement de certains instruments dans leurs propres locaux contrairement aux recommandations des BPR. Cette attitude était à l´origine de pratiques inadéquates à l´instar de l´utilisation des tambours à éclipses comme moyen de conditionnement. D´autre part, la définition des responsabilités d´un pharmacien à la tête de l´unité, ayant des compétences en matière de matério-vigilance et de management de la qualité, expliquait le taux le plus élevé (60%) de la rubrique «maitrise des dispositifs de surveillance et de mesure» dans le rapport du premier audit.

Les points faibles de l´unité étaient liés à des défaillances organisationnelles et attribuées principalement à l´absence d´un SMQ, aux dysfonctionnements des équipements, aux anomalies architecturales des locaux et aux compétences limitées des ressources humaines. Ces constats rejoignaient ceux décrits et mentionnés dans l´enquête de l´ANCSEP en 2011 [3]. Etant donné que le taux conformité global déterminé par le premier audit était limité à 28%, la situation initiale était éloignée du niveau de sécurité requis pour le retraitement des DMR. Depuis la parution de la nouvelle version de la norme NF EN ISO 14385, la gestion des risques est devenue une composante essentielle du SMQ des unités retraitant les DMR [9]. Dans ce travail, l´audit a révélé qu´aucune analyse des risques relatifs au processus de retraitement des DMR n´a été jusque-là établie. Bien que cette défaillance ne représente pas une déviation de point de vue règlementaire, l´absence d´une telle étude était considérée comme une inconvenance majeure par les BPR. En effet, ces dernières exigeaient l´évaluation des risques et la mise en place des mesures permettant de les maitriser selon les principes de la norme NF EN ISO 14971 [10]. L´intérêt de cette approche dans l´optimisation et la maitrise du circuit des DMR a été confirmé par de multiples auteurs [11].

Le personnel de stérilisation occupe une place prépondérante dans le circuit de retraitement des dispositifs médicaux vu son implication dans les différentes étapes du processus [12]. Ainsi, la circulaire N°8 de l´année 2015 recommande l´élaboration d´un organigramme opérationnel et des fiches de fonctions pour chaque agent de l´unité. Ces derniers devraient bénéficier de formations initiales et continues en stérilisation. Dans notre étude, le taux de conformité de la sous rubrique «ressources humaines» était nul en absence d´un organigramme et des cahiers de charges relatifs aux différents postes. Les agents de stérilisation avaient des formations initiales peu adaptées à la stérilisation et leurs connaissances théoriques et pratiques ont été transmises oralement dès leurs intégrations. Ce défaut en formation spécialisée prédisposait au risque d´erreur et de pratiques aberrantes entravant la qualité des services fournis. En effet, Fast *et al*. [13] ont montré que le manque d´une formation spécialisée en stérilisation était la cause principale des défaillances majeures identifiées lors d´une évaluation du processus de stérilisation dans 59 établissements de soins dans 3 pays à revenu limité.

Concernant les ressources de l´unité, des déviations par rapport aux standards ont été aussi identifiées. En effet, le rapport d´audit a dévoilé des anomalies architecturales et un taux de conformité des locaux égal à 18%. La conception de l´unité ne permettait pas de respecter le principe de la marche en avant. Cette défaillance augmente de manière critique le risque de confusion entre les DMR stériles et ceux non stériles [14]. Bien que l´eau soit la ressource la plus critique en stérilisation, le taux relatif à cette rubrique était nul. Sa qualité conditionne non seulement l´efficacité de la stérilisation, mais aussi la pérennité des équipements (laveur désinfecteur et autoclave) ainsi que celle des DMR stérilisés [15]. L´air médical comprimé, quant à lui, est utilisé principalement dans le séchage des DMR. Les éventuelles impuretés potentiellement présentes dans ce fluide pourraient constituer une source de contamination pour les instruments préalablement nettoyés. Pour cette raison, les BPR recommandent le contrôle de sa qualité en se basant sur les exigences spécifiques de la norme ISO 11137-3 [7]. Ainsi, l´air médical devrait satisfaire aux exigences de la classe de pureté 2 spécifiée par la norme ISO 8573-1 [16]. Dans notre étude, l´audit a rapporté que la qualité de l´air médical comprimé utilisé dans l´unité de stérilisation n´avait jamais été contrôlée.

Concernant les processus, nous avons identifié des dysfonctionnements qui pourraient avoir un impact important sur le retraitement des dispositifs médicaux. Le taux de conformité pour cette rubrique était de 29,5%. En absence d´un laveur désinfecteur, l´étape de nettoyage n´a pas été évaluée de façon exhaustive. Tous les DMR subissaient un nettoyage manuel pourtant cette technique n´est plus recommandée par la norme NF EN ISO 15883-2, car elle est non standardisée et peu reproductible [17]. D´autre part, l´audit a révélé une irrégularité des contrôles de la propreté et de la fonctionnalité des DMR. Cette faiblesse était due au défaut des moyens de contrôle (loupe, microscope, instruments gainés, optique) et au manque de formation des agents de stérilisation sur ce sujet.

Pour l´étape de conditionnement, le taux de conformité était limité à 10%. Premièrement, cette étape n´était pas validée conformément aux exigences de la norme ISO 11607 [18]. Par ailleurs, l´absence d´un contrôle quotidien de la thermo-soudeuse ainsi que l´entretien irrégulier du parc des conteneurs pourrait expliquer les problèmes d´étanchéité des charges stérilisés. De même, en l´absence de fiches de composition des plateaux opératoires, des incidents de perte ou de permutation des instruments ont été signalés. De tels incidents ont été également rapportés par Zhu *et al*. [19] au cours d´une étude observationnelle sur les facteurs impliqués dans erreurs de conditionnement des instruments chirurgicaux.

Pour l´étape de stérilisation proprement dite, notre étude a révélé un taux de conformité de 36%. L´autoclavage en absence de dossiers de charges était le principal écart enregistré. En outre, les paramètres physiques relatifs aux cycles (température, pression, temps d´exposition) étaient non tracés. Pourtant, une étude multicentrique, en comparant les résultats des tests chimiques et les graphiques des cycles correspondants, a montré que les cycles étudiés présentaient au moins un paramètre non conforme aux exigences malgré un test chimique conforme [20].

L´état de l´unité de stérilisation siège de l´étude ne faisait pas l´exception. Des situations similaires ont été décrites. Bien que certains dysfonctionnements ont été rapportés dans des pays développés [21], la situation dans les pays en cours de développement était plus critique [22]. Le rapport du premier audit était un motif intéressant pour établir l´état des lieux dans l´unité de stérilisation. Les actions d´amélioration entreprises ont porté principalement sur la mise en place d´un SMQ et la formation du personnel. En effet, cette dernière était indispensable non seulement pour garantir leur adhésion à la démarche suivie, mais également pour les sensibiliser à l´importance de l´assurance qualité et notamment l´intérêt de la traçabilité dans la mise en valeur de leurs activités. L´impact de la formation sur la prise en charge des DMR dans les unités de stérilisation a été prouvé par les études de Fast et ses collaborateurs dans 15 hôpitaux en Afrique [23, 24]. Toutefois, les auteurs recommandaient une réévaluation et un entretien régulier des connaissances acquises. Dans le même contexte, le responsable de l´unité pourrait s´inspirer de l´expérience des hôpitaux français qui ont développé de nouveaux outils pédagogiques pour la formation des agents de stérilisation [25].

Le deuxième audit a été réalisé pour évaluer l´impact des actions d´amélioration mises en œuvre au sein de l´unité. Les résultats obtenus ont montré une augmentation du taux de conformité global de 32,3% (60,4% pour le 2^e^ audit contre 28,1% au 1^er^ audit). Toutefois, cette amélioration était insuffisante pour atteindre le niveau de sécurité escompté. Même si le taux de conformité de la rubrique «retraitement des dispositifs médicaux» avait haussé de 18% (47,5% pour le 2^e^ audit contre seulement 29,5% au 1^er^ audit), l´unité continuait à présenter des défaillances spécifiques souvent qui échappaient à ses contrôles. En effet, l´absence d´une collaboration effective des services clients notamment les blocs opératoires avait entravé la mise en place d´une série de mesures telles que le recensement du parc d´instruments et la mise en place d´une procédure de gestion des instruments en prêts. Dans le même cadre, une enquête menée en France et portant sur l´analyse du circuit des ancillaires en milieu hospitalier, a rapporté la complexité de ce circuit au nombre des acteurs intervenant et à la variabilité de ces DMR [26]. Les auteurs recommandaient dans ce cas une concertation et une collaboration plus active entre les différents acteurs afin de définir et répartir les tâches.

L´analyse des résultats du deuxième audit a permis également d´identifier des opportunités d´amélioration notamment l´informatisation du circuit des DMR et l´application de nouveaux concepts de management dans l´unité. À cet effet, le développement d´une traçabilité à l´instrument représente une piste intéressante. Elle consiste à tracer individuellement les instruments par l´intermédiaire de codes «Datamatrix» collés ou gravés ou des puces RFID (*radio frequency identification*) y sont insérées [27]. Cette procédure permettait une meilleure prise en charge des plateaux opératoires notamment pendant l´étape de conditionnement puisqu´à chaque conteneur, serait associée une composition bien déterminée. D´autre part, le recours à la gestion de la production Lean ou «le Lean management» constitue un levier d´amélioration. Cette méthode est de plus en plus fréquente dans le milieu hospitalier et particulièrement en stérilisation [28]. Appliquée dans un centre médical en Virginie aux Etats unis, elle a permis de réduire de manière significative les erreurs liées à la recomposition des sets [29]. De même, son adoption dans un hôpital à Montpellier a permis la fluidification de l´activité de recomposition et la réduction du temps de nécessaire au retraitement d´un plateau opératoire de 44 à 30 heures [30]. Enfin, cette méthode présente un intérêt économique. En effet, Alves *et al*. [31] ont montré que le coût de stérilisation d´un plateau a passé de 134,25 à 40,2 euros en utilisant le lean management comme outil d´optimisation de la composition des plateaux opératoire.

Au terme de ce travail, nous avons répondu aux objectifs préalablement fixés. Toutefois, notre étude présentait quelques limites. Tout d´abord, nous avons adopté l´audit interne pour évaluer le processus de stérilisation dans l´hôpital. Cette approche pourrait manquer d´objectivité. D´autre part, l´étape de pré-désinfection n´a pas été prise en compte durant toute l´étude. Un éventuel audit supplémentaire devrait remédier à cette anomalie. De même, les nombres élevés des critères non applicables (72 et 63 pour le premier et le deuxième audit respectivement) reflétaient que cette grille était relativement adaptée à notre étude. Enfin, ce travail devrait être complété par une analyse des risques pour pouvoir les hiérarchiser en fonction de leur criticité. Ainsi, les actions à entreprendre seraient mieux efficientes.

## Conclusion

L´amélioration du taux de conformité global de 28,1% lors du premier audit à 60,4% à la fin du deuxième audit témoignait les efforts déployés par toute l´équipe participant à la mise à niveau de cette unité. Les actions entreprises ont porté principalement sur l´élaboration du système documentaire et la mise en place du SMQ. Cette stratégie, qui a permis la standardisation des pratiques et la sécurisation du circuit des DMR, avait un impact positif sur les relations entre l´unité et les services clients. Elle a également créé une dynamique non seulement au sein de l´unité de stérilisation, mais aussi pour les décideurs de l´établissement. Afin de répondre aux exigences règlementaires et normatives, toutes les parties intervenantes devraient être plus engagées dans la prise de décision et l´application des actions d´amélioration.
